# Isolation and Characterization of Broad Spectrum Coaggregating Bacteria from Different Water Systems for Potential Use in Bioaugmentation

**DOI:** 10.1371/journal.pone.0094220

**Published:** 2014-04-15

**Authors:** Zhongqin Cheng, Xiangxun Meng, Haichao Wang, Mei Chen, Mengying Li

**Affiliations:** School of Biology and Basic Medical Sciences, Soochow University, Suzhou, PR China; Loyola University Medical Center, United States of America

## Abstract

The bridging bacteria with broad-spectrum coaggregation ability play an important role during multispecies-biofilm development. In this study, through a visual and semi-quantitative assay, twenty-two bacterial strains with aggregation ability were obtained from 8 different water environments, and these strains were assigned to 7 genera according to their 16S rDNA and they were *Aeromonas*, *Bacillus*, *Comamonas*, *Exiguobacterium*, *Pseudomonas*, *Shewanella* and *Comamonas*. Furthermore, all possible 231 pairwise combinations among these 22 strains were explored for coaggregation ability by spectrophotometric assay. Among all these strains, it was found that *Bacillus cereus* G5 and *Bacillus megaterium* T1 coaggregated with themajority of assayed other strains, 90.5% (19 of 21 strains) and 76.2% respectively (17 of 21 strains) at a higher coaggregation rates (A.I. greater than 50%), indicating they have a broad-spectrum coaggregation property. The images of coaggregates also confirmed the coexistence of G5 and T1 with their partner strains. Biofilm biomass development of G5 cocultured with each of its partner strains were further evaluateded. The results showed that 15 of 21 strains, when paired with G5, developed greater biofilm biomass than the monocultures. Furthermore, the images from both fluorescence microscopy and scanning electron microscopy (SEM) demonstrated that G5 and A3-GFP (a 3,5-dinitrobenzoic acid-degrading strain, staining with gfp),could develop a typical spatial structure of dual-species biofilm when cocultured. These results suggested that bridging-bacteria with a broad spectrum coaggregating ability, such as G5,could mediate the integration of exogenous degrading bacteria into biofilms and contribute to the bioaugmentation treatment.

## Introduction

Coaggregation is a process by which genetically distinct bacteria become attached to one another via specific molecules. It is proposed that coaggregation influences biofilm formation and species diversity during the development of complex multispecies biofilms [1], [2]. Numerous researchers have demonstrated that coaggregation could occur among multiple bacterial species and is a common phenomenon between widespread bacterial genera to different extents [3]–[5]. The earliest and extensive studies assessing the ability of bacterial cells to recognize and communicate with one another leading to coaggregation, primarily focused on oral biofilms or dental plaques. More than 1000 bacterial strains from the oral cavity were demonstrated to be of coaggregation ability [6], [7].

Among them, *Fusobacterium nucleatum* displayed a broad spectrum coaggregation ability. *F. nucleatum* coaggregated with multiple bacterial species, enhancing the development of biofilms in the oral cavity and mediating the integration of pathogens into biofilms [6], [8].

There are many biofilm communities in other ecosystems besides oral biofilms [9]. In recent years, biofilms microflora presented in both natural and wastewater-treated environments were explored, and several new strains with bridging function were found [2], [5], [10]–[13]. But those studies mainly focused on screening bacterial species with coaggregation ability and on coaggregation mechanisms [8], [14]–[16]. Studies on potential applications of these bacteria are very limited.

Bioaugmentation, the introduction of new metabolic functions by the addition of bacteria, is a possible way for the degradation of recalcitrant xenobiotic compounds. A major obstacle to successful bioaugmentation is the washaway of inoculated microbes. The colonization and survival of inoculated microbes are very critical for successful bioaugmentation in wastewater treatment. Researchers tried to solve this problem by adding a series of fixatives such as Calcium alginate [17], PVA [18], chitosan [19], etc, for degrading-bacteria inoculum persistence in treatment systems. However, these methods may be cost prohibitive or troublesome to perform. In contrast, biofilms, as native forms of cell immobilization, inherently display advantages in tolerance to hostile environments, as well as proliferation of the bacteria immobilized [20]. Consequently, it would be a simple and inexpensive way for the integration of degrading bacteria into biofilms of wastewater treatment systems by bridging those bacteria with a broad spectrum coaggregating ability.

This work investigated the coaggregation ability and biofilm-forming ability of selected bacteria isolated from several ecosystems and explored the potential of the integration of degrading bacteria into biofilms through coaggregation interactions.

## Materials and Methods

### Ethics Statement

No specific permits were required for the described field studies. No specific permissions were required for these locations. We confirm that the location is not privately-owned or protected in any way. We confirm that the field studies did not involve endangered or protected species.

### 2.1 Identification of Bacterial isolates

Biofilms collected from municipal sewage, stones in landscape ponds and the industrial wastewater treatment system were incubated with 5% or 10% LB (v:v) solution to isolate the bacterial species. Individual colonies were selected according to morphology. After purification using the streaking method, isolates were selected and stored at −80°C for future use. Isolates were identified by Gram staining and standard physiological and biochemical tests and were further characterized by 16S rDNA sequencing.

The other bacterial strains used in the following experiments were maintained in Microorganism Laboratory of Soochow University. *Pseudomonas sp.M8, Pseudomonas putida M9, Aeromonas cavia M10, Pseudomonas plecoglossicida M21, Aeromonas hydrophila M22* and *Comamonas testosterone* A3 were obtained from the biofilms of a nitrobenzene wastewater treatment system [21], *Pseudomonas aeruginosa* XCZ from a petroleum and chemical wastewater treatment system [22] and *Pseudomonas putida* DLL from an agricultural chemical wastewater treatment system [23].

### 2.2 Scanning electron microscopy (SEM)

The sample for SEM was washed and hardened by suspending in a 2.5% glutaraldehyde solution for 1 h. Subsequently, the sample was dehydrated in a series (20%, 40%, 60%, 80% and 99.5%) of ethanol solutions for 30 min each. Subsequently, the pellet was suspended in 3-methylbutyl acetate. After 12 h critical point drying was performed using a critical point drier (Hitachi HCP-2). Then, the sample was fixed and scanned under a scanning electron microscope (Hitachi S-4500).

### 2.3 Preparation of bacterial suspensions

Stored strains were cultured on solid LB medium at 30°C for 18–24 h to be activated. Flasks (250 ml) containing 100 ml liquid LB medium were inoculated with activated cells at 30°C for 24 h at 180 rpm. Cells were harvested by centrifugation at 5,000 rpm for 5 min, washed twice in PBS buffer (0.1 mol/L, pH 7.0), and resuspended in the same solution. Finally, the optical density (OD_660_) of the cellular suspension concentration was adjusted to 1.0 for future use.

### 2.4 Coaggregation assay

Coaggregation rates were determined by a modified method described by Malik et al.[13]. Cell suspensions (OD_660_ = 1.0) of bacterial partners were prepared as described above. Equal volumes (5 ml) of coaggregating partner suspensions were mixed in 15-ml centrifuge tubes and incubated at 20°C. Pure bacterial suspensions (10 ml) were incubated under the same conditions as controls. Samples were cultured for 2 h or 20 h,subsequently centrifuged at 500 rpm for 1 min, then the OD_660_ of the supernatant were measured.

The coaggregation index (A.I.), representing the coaggregation rate (%), was calculated as follows: 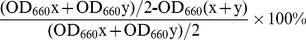
where OD_660_x and OD_660_y represent the optical density at 660 mm for each of the individual pure-cultures and OD_660_(x+y) represents the optical density for the mixtures [3].

Rapid evaluation of the coaggregation rate was performed by a visual and semi-quantitative assay described by Rickard et al. [24] with modification [25].

### 2.5 Quantification of biofilm biomass

Tubes (10×75 mm) containing 2 ml 5% LB solution were inoculated with 2 µl previously prepared bacterial cell suspensions (half volume of each in the case of the 2 bacterial strains), and the liquid cultures were incubated at 30°C at 150 rpm for 20 h. Biofilm quantification was performed using a crystal violet stain described previously by Zhu et al. [26]. Briefly, after 20 h of incubation, the medium containing suspended cells was removed and the tubes rinsed twice with distilled water, and the remaining attached biomass were stained for 30 min with 50 mL 0.1% (w/v) crystal violet in water. The tubes were washed thoroughly with water and dried overnight. The retained crystal violet was dissolved in 10 mL of ethanol-acetone (80: 20 v/v), and absorbance (OD_570_ nm) was measured at 570 nm.

### 2.6 Observation of biofilm structure

Biofilm development was performed with slides as described previously by Bechet M et al.[27] and Tait K et al.[28]. Two slides (24.5×76.2×0.8 mm) were added to 150 ml flasks containing 50 ml 5% LB liquid to serve as biofilm development devices. The devices were inoculated with previously prepared 500 uLbacterial suspensions (250 uLfor each in the case of the 2 bacterial strains) and incubated at 100 rpm at 30°C for 12 h. *Comamonas testosterone* A3 was transfected with a GFP plasmid through a method described by Kerry et al. [29]. The GFP expression plasmid pTRGFP (Amp^r^, Tc^r^) is a broad host vector, constructed in the Key Laboratory of Microbiological Engineering of Agricultural Environment lab [23]. pTRGFP in *E. Coli* DH5α was introduced into A3 (Spe^r^) by triparental mating, where the plasmid transfer function was provided *in trans* by *E. coli* HB101 (Km^r^, a triparental mating helper strain containing pRK2013). The resulting mated cells were screened on LB agar containing 100 µg/ml spectinomycin to exclude *E. coli* and 100 µg/ml ampicillin and 20 µg/ml tetracycline to select for A3 cells with pTRGFP. The tagged isolate was referred to as *C. testosterone* A3/GFP (A3/GFP).

The slides were subjected to imaging using a live cell imaging system (Olympus, Cell'R) and a scanning electron microscope (Hitachi S-4500) according to the standard protocols.

## Results

### 3.1 Preliminary screening and identification of coaggregated bacteria in different ecological systems

Ten bacterial strains were isolated from biofilms in the pollutant water treatment pool of Wujiang Longying dyeing mill and were mixed together in pairs to compare their coaggregation ability by visual observation [3]. Two of them, F2 and F3, showed better coaggregating ability (results not shown). Following the same steps, G3, G5 and G6, were obtained among 14 isolates from biofilms at the domestic sewage sewer outlay; I1, I2, N2 and Q2 among 15 isolates from biofilms of a municipal wastewater treatment plant at the east of Suzhou city; H1, H2 and H3, among 12 isolates from the west landscape pond at the Campus of Soochow University; T1 and T2, among 9 isolates from the east landscape pond at the Campus of Soochow University. In total, 14 bacterial strains were isolated in this screening experiment. Their morphological, physiological, and biochemical characteristics and the base length of the sequenced 16S rRNA genes are presented in Table 1. The 14 bacterial strains were assigned to 6 genera or species. Of the 14 strains, seven belonged to *Bacillus spp.*, two *Shewanella cinica*, two *Aeromonas spp.*, one *Pseudomonas spp*, one *Shewanella oneidensis* and one *Exiguobacterium sp.* These coaggregating bacteria belong to multiple genera, demonstrating the wide occurrence of this property in environmental microflora.

**Table 1:** The characterization and identification of bacterial isolates by sequencing of 16S rRNA genes.

Isolate	Accession no. Of sequence in this study	% Identity of base sequences	16SrDNA Sequence length(bp)	Proposed identity	Cell morphology	Result of Gram staining	Presence of oxidase	Presence of catalase
F2	HQ418485	99.9	1400	*Pseudomonas* sp.	Small rods	–	+	+
F3	HQ418486	99.7	1394	*Alishewanella* sp.	Small rods	–	–	–
G3	HQ418487	99.8	1445	*Bacillus* sp.	Large rods	+	+	–
G5	HQ418488	99.8	1422	*Bacillus cereus*	Large rods	+	+	+
G6	HQ418489	99.7	1459	*Bacillus* sp.	Large rods	+	+	+
H1	HQ418491	99.9	1428	*Bacillus* sp.	Large rods	–	–	–
H2	HQ418492	99.5	1446	*Shewanella* sp.	Small rods	–	+	–
H3	HQ418493	99.5	1445	*Shewanella* sp.	Small rods	–	+	–
I1	HQ418494	94.6	1486	*Bacillus cereus*	Large rods	+	+	+
I2	HQ418495	99.8	1440	*Exiguobacterium* sp.	Small rods	–	–	+
N2	HQ418496	99.7	1001	*Aeromonas* sp.	Small rods	–	+	+
Q2	HQ418497	98.7	1429	*Aeromonas* sp.	Small rods	–	+	+
T1	HQ418498	99.6	1432	*Bacillus megaterium*	Large rods	+	+	+
T2	HQ418499	93.9	1477	*Bacillus* sp.	Large rods	+	+	–

### 3.2 Coaggregation ability of bacteria from various ecological environments

**Figure 1:**
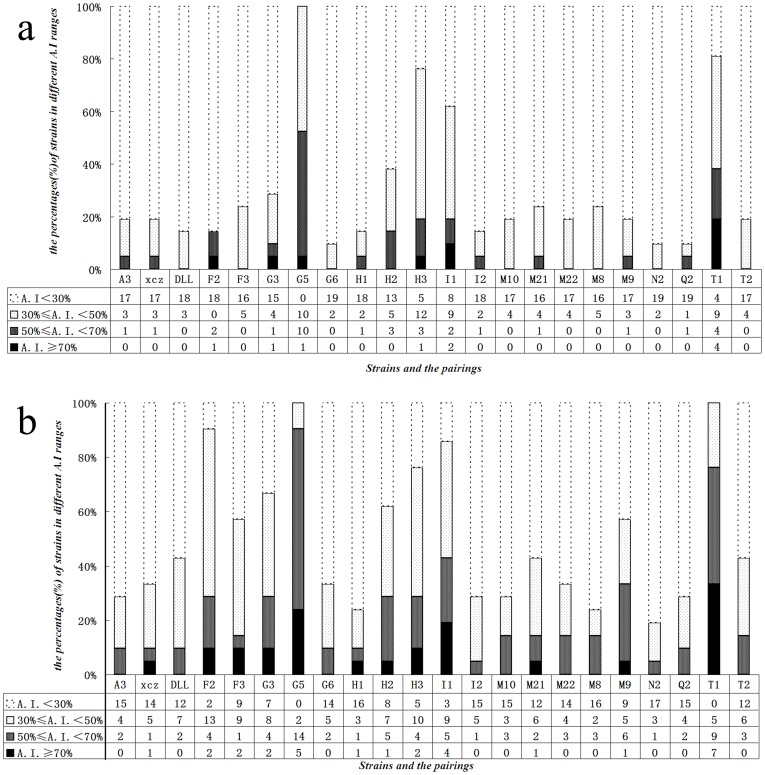
Numbers of strains pairings and their percentages for each of the 22 strains at 2 h (a) and 20 h (b) in different A.I. ranges (A.I.: coaggregation index). The bacteria were incubated at 20°C for 2 h or 20 h.

These 14 strains together with 8 other strains stored in Microorganism Laboratory of Soochow University were surveyed for coaggregating ability. All 231 possible pairwise combinations of 22 strains were assessed for coaggregation ability by spectrophotometry. Of all 231 pairwise combinations, 42 pairs showed >50% coaggregation rates after a 2 h cocultivation period, and 125 pairs exhibited >50% coaggregation rates after a 20 h cocultivation period (Table S1, Table S2, Fig. 1a, Fig. 1b), indicating that coaggregating ability increased as time prolonging.

Although all 22 strains displayed coaggregation ability during preliminary screening their coaggregating ability, with respect to numbers and extensiveness of partners, varied from strain to strain. As shown in the coaggregation matrix (Table S2), even after a 20 h cultivation period, I1 and N2 could only coaggregate with one partner strain, exhibiting >50% coaggregation rates; A3, XCZ, DLL, G6, H1 and Q2 could coaggregate with two partner strains; F3, M21, M22, M8, M10 and T2 with three partner strains; F2, G3, H2 and H3 with six; and I1 with nine.

Among all assayed strains, G5 and T1 showed the strongest coaggregation ability. G5 could coaggregate with 19 partner strains (90.5%) belonging to 7 bacteria genera at high coaggregation rate(>50%)after a 20 h cultivation period, while T1 with 16 strains (76.2%) of 6 bacteria genera(Fig. 1). Furthermore, both G5, coaggregating with 5 partner strains and T1, with 7 partner strains, exhibited greater than 70% coaggregation rates. These results indicated that G5 and T1were highly efficient coaggregators that can coaggregate with most of the remaining 21 strains, and displayed a broad-spectrum coaggregation ability.

**Figure 2:**
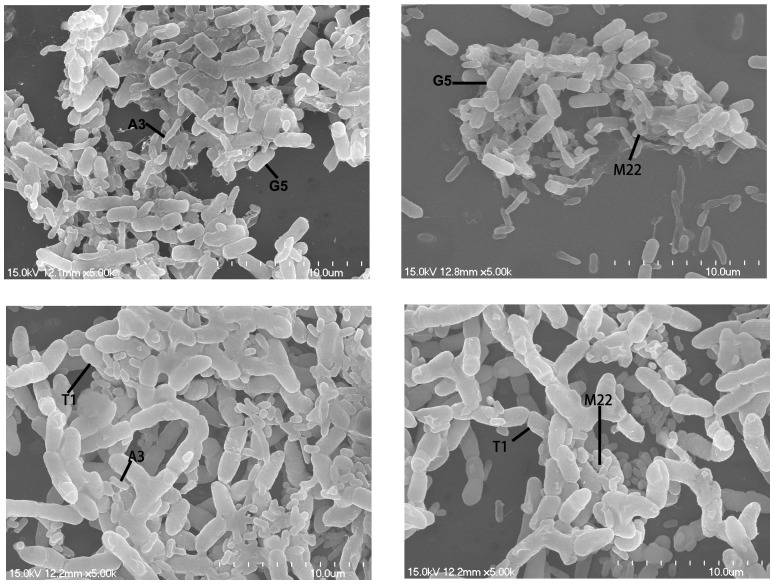
Scanning electron micrograph of the coaggregates. a: G5 with A3; b: G5 with M22; c: T1 with A3; d: T1 with M22; Larger G5 and T1 cells were surrounded by many smaller bacterial cells (A3 or M22), showing stable coaggregate mixtures.

The structures of coaggregate mixtures of selective pairs, including G5 with A3, G5 with M22, T1 with A3 and T1 with M22, were further observed by scanning electron microscopy. Images (Fig. 2) revealed that larger G5 and T1 cells were surrounded by smaller bacterial cells, A3 or M22, showing stable coaggregate mixtures.

### 3.3 Biofilm formation ability for G5

Since G5 performed a broard spectrum coaggregating ability and highlighted a possible role as a bridging organism in the biofilm consortium, it was tested for the biofilm-forming ability subsequently.

**Figure 3:**
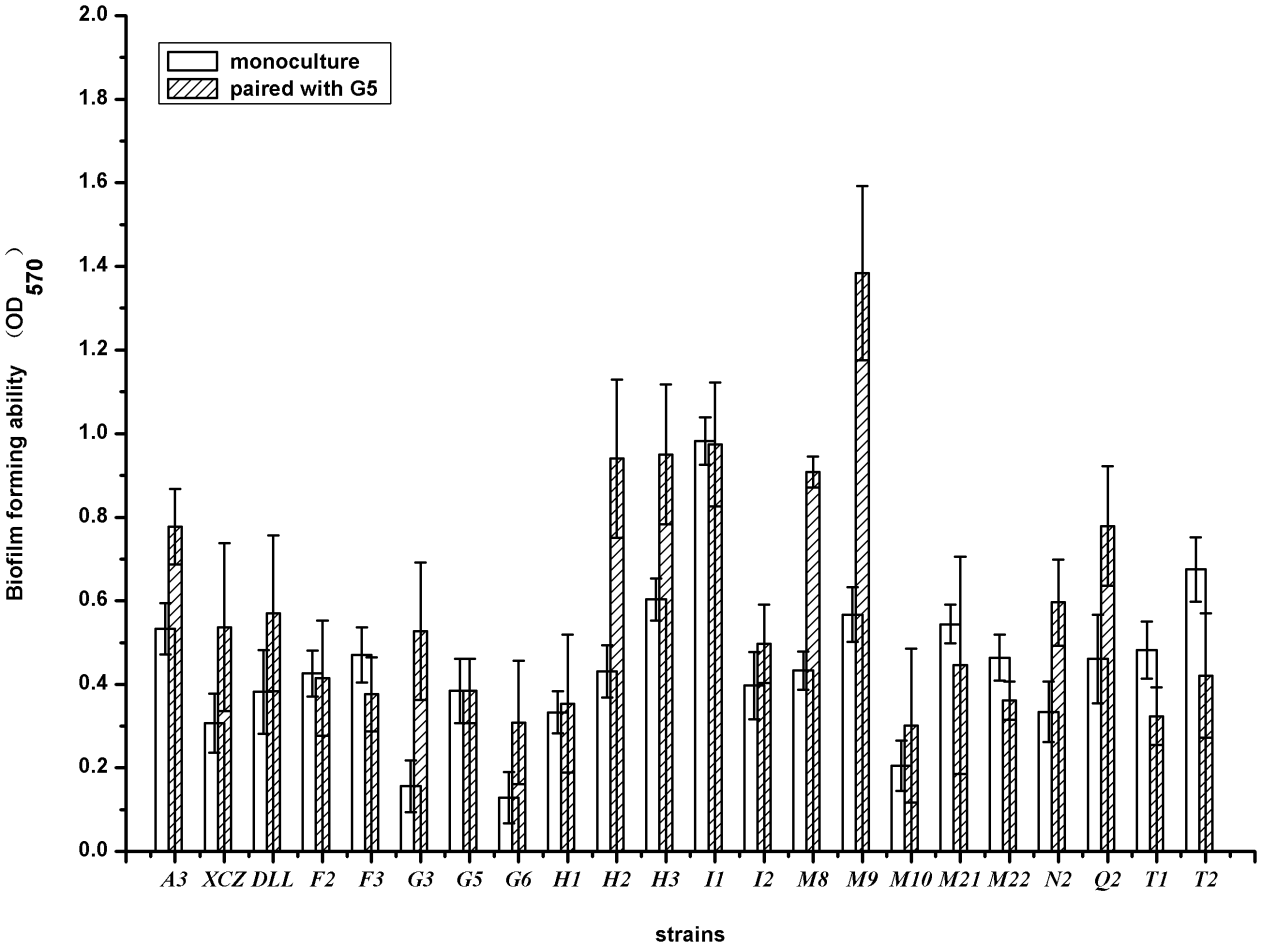
Biofilm biomass compare of G5 with different strains. The bacteria were incubated at 30°C at 150 rpm for 20 h in 5% LB. The error bars represent ± SD of the assay performed in triplicate.

Biofilm biomass from coaggregating mixtures of G5 with each of the 21 other strains, along with their monocultures, were quantified under low nutrient conditions (5% LB). The results showed that coaggregating pairs of G5 with 15 of tested 21 strains (71.4%) produced greater biofilm biomass than any monoculture (Fig. 3). The t-test results demonstrated that the calculated t value was 45.642 when comparing the G5 cocultured means with monocultured means, which was significantly higher than 2.704 at t_0.01_ (df = 40), indicating that the cocultured G5 biofilm biomass increased significantly even at this low nutritional conditions.

Interestingly, in all of these 15 pairs, three pairwise combinations between G5 and degrading bacteria (A3, XCZ and DLL) showed greater biofilm biomass than the monoculture (Fig. 3), indicating that G5 could cooperate with these exogenous degrading bacteria and develop more biofilm biomass effectively.

### 3.4 Degrading bacteria A3 immobilization mediated by G5

**Figure 4:**
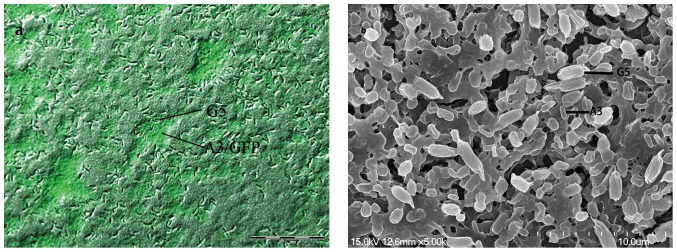
Fluorescence microimages (a) and Scanning electron micrograph (b) of G5-A3/GFP biofilms. Biofilms were developed on slide surfaces and were visualized with FM and SEM using G5 and A3/GFP (expressing GFP). Two different sizes bacteria were observed in the structural biofilms.

To obtain more detailed information about coaggregation ability of G5, biofilms from G5-A3/GFP were cultured on slides and analyzed. Under fluorescence microscope, biofilms were shown green and dense, consisting of two different sizes of bacteria (Fig. 4a). To obtain higher resolution images, similar biofilms were analyzed by SEM. Again, cell coaggregates were evident and two different sizes of bacteria were observed. In addition, extracellular material was apparent in biofilms under high resolution SEM (Fig. 4b). The images confirmed that A3 was integrated into the biofilm, and G5 with A3/GFP constructed structural and heterogeneous biofilms. These results demonstrated that G5, with its coaggregating ability, could immobilize degrading bacteria into biofilms effectively.

## Discussion

### 4.1 Bacterial species and genera with broad-spectrum coaggregating ability

Screening of bacteria with coaggregation ability and exploration of such microbial resources may have important significance in bioaugmentation treatment. Simões et al. [2] demonstrated that the bacterium *Acinetobacter calcoacticus* from drinking water biofilms displayed not only an autoaggregation ability but also coaggregated with 4 other isolates in a biofilm-dependent manner. Buswell et al. [3] isolated 19 strains from biofilms in artificial cultivation water and suggested *Micrococcus luteus*, as a bridge, could coaggregate with 11 strains. Malik et al. [13] isolated 32 bacterial strains from sewage-activated sludge and observed that *Acinetobacter johnsonii* S35 coaggregated with 7 bacterial genera including 32 isolates. These coaggregating bacteria belonged to multiple genera, demonstrating a diversified existence of such ability within bacteria.

In this study, sixty bacterial isolates were isolated from biofilms of various ecological water environments. Thereafter fourteen of these strains showing strongly coaggregating ability were obtained by a preliminary visual screen. In addition, eight previously isolated strains with strong biofilm formation ability or certain biodegrading ability were obtained from the Microbiology Laboratory of Soochow University. In total, all 22 bacterial strains were used in further experiments and were assigned to 7 genera. Among these 22 strains, only two, G5 and T1, were able to coaggregate with multiple species and exhibited the strongest coaggregation ability. G5 could coaggregate with 19 partner strains (90.5%) at high coaggregation rate(>50%)after a 20 h cultivation period, while T1 with 16 strains (76.2%) (Fig. 1). Furthermore, both G5, coaggregating with 5 partner strains and T1, with 7 partner strains, exhibited greater than 70% coaggregation rates.

These two bacterial strains, i.e. G5 and T1, that manifested wide coaggregation abilities, belonged to bacillus genus, which occupies various natural water and wastewater treatment systems. Previously, many *Bacillus* strains had been found to be of coaggregating ability. Two independent studies reported that both *Bacillus thuringiensis* I2 and *Bacillus* sp. VA160, isolated from sledge particles, could coaggregate with 2 other strains [11], [30]. Rickard et al. [1] suggested that 4 *Bacillus* strains could coaggregate with 4–9 strains of 28 isolates, demonstrating a coaggreggation ability. Nevertheless, there are few reports that identify that the *Bacillus* strains harbor a broad coaggregation ability and also participated in bridging.

### 4.2 Widely coaggregating bacterial function in bioaugmentation

It was known that immobilization of degrading-bacteria was a key for successful wastewater treatment in the bioaugmentation system. Biofilms, as natural forms of cell immobilization in wastewater treatment systems, have advantages over other approaches, such as tolerance to hostile environments, proliferation of the bacteria immobilized and the low cost of immobilization. The immobilization of degrading-bacteria in the biofilms has been suggested as a strategy for maintaining efficient degradation in the bioaugmentation system [31]. Recently Yunyoung K wak et al. [12] reported that *Sphingomonas* sp. 224 co-inoculated with biofilm-forming bacterium *Pseudomonas* sp. C7 and *Bacillus* sp. E5. was observed to degrade tolclofos-methyl more effectively than *Sphingomonas* sp. 224 alone. Some researchers have indicated that *Comamonas* sp. PG-08, via its aggregation ability but not the phenol-degrading ability, enhanced the ability of *Propioniferax*-like PG-02 to degrade phenol [32]. Additionally, *Bacillus thuringiensis* I2 and *Bacillus* sp.VA160 could promote degradation efficiency of another strain via their coaggregation but not degradation ability [11], [30]. In the present study, A3 strain, degrading nitrobenze, were cocultured with G5 to explore this immobilization for the further usage in bioaugmentation. A typical biofilm with spatial heterogeneity structure was observed with fluorescence microscope and SEM, and degrading bacteria were immobilized into the biofilm effectively. This immobilization of bacterial species in wastewater treatment could be simple, affordable, and stable in comparison to artificial immobilization techniques that rely on embedding agents. Hence these broad spectrum coaggregating strains, such as G5, could be applied in bioaugmentation systems for an effective performance.

## Supporting Information

Table S1 **Coaggregation index(A.I.) for bacterial pairs from 22 strains at 2 h.** The bacteria were incubated at 20°C; the OD_660_ of the supernatant were measured. The values are the average of three independent experiments with deviation score in brackets.
(DOCX)

Table S2 **Coaggregation index (A.I.) for bacterial pairs from 22 strains at 20 h.** The bacteria were incubated at 20°C; the OD_660_ of the supernatant were measured. The values are the average of three independent experiments with deviation score in brackets.
(DOCX)
