# Supplementary material for: Isolation and Characterization of Broad Spectrum Coaggregating Bacteria from Different Water Systems for Potential Use in Bioaugmentation
Source: PLoS One. 2014 Apr 15;9(4):e94220. doi: 10.1371/journal.pone.0094220 (PMC3988075; doi:10.1371/journal.pone.0094220)
Supplement: Table S1 — Coaggregation index(A.I.) for bacterial pairs from 22 strains at 2 h. The bacteria were incubated at 20°C; the OD660 of the supernatant were measured. The values are the average of three independent experiments with deviation score in brackets. (DOCX) [file pone.0094220.s001.docx]

**Table S1 Coaggregation index(A.I) for bacterial pairs from 22 strains at 2 h**

| **Strain** | | | | | | | | | | | | | | | | | | | | | | |
| --- | --- | --- | --- | --- | --- | --- | --- | --- | --- | --- | --- | --- | --- | --- | --- | --- | --- | --- | --- | --- | --- | --- |
| **Strain** | **A3** | **XCZ** | **DLL** | **F2** | **F3** | **G3** | **G5** | **G6** | **H1** | **H2** | **H3** | **I1** | **I2** | **M8** | **M9** | **M10** | **M21** | **M22** | **N2** | **Q2** | **T1** | **T2** |
| **A3** |  |  |  |  |  |  |  |  |  |  |  |  |  |  |  |  |  |  |  |  |  |  |
| **XCZ** | 12.5±5.5 |  |  |  |  |  |  |  |  |  |  |  |  |  |  |  |  |  |  |  |  |  |
| **DLL** | 12.4±4.6 | 15.9±3.4 |  |  |  |  |  |  |  |  |  |  |  |  |  |  |  |  |  |  |  |  |
| **F2** | 16.5±6.4 | 15.6±5.3 | 15.6±6.4 |  |  |  |  |  |  |  |  |  |  |  |  |  |  |  |  |  |  |  |
| **F3** | 20.8±4.6 | 32.8±4.5 | 20.3±5.5 | 13.9±3.3 |  |  |  |  |  |  |  |  |  |  |  |  |  |  |  |  |  |  |
| **G3** | 23.9±5.7 | 36.1±6.9 | 25.2±7.2 | 13.9±4.5 | 21.8±7.4 |  |  |  |  |  |  |  |  |  |  |  |  |  |  |  |  |  |
| **G5** | 52.2±8.5 | 57.9±6.3 | 35.7±5.3 | 52.4±7.4 | 40.9±6.5 | 54.3±7.7 |  |  |  |  |  |  |  |  |  |  |  |  |  |  |  |  |
| **G6** | 13.6±4.3 | 15.3±4.5 | 17.5±3.5 | 16.3±3.4 | 10.8±3.7 | 24.2±5.8 | 39.4±4.5 |  |  |  |  |  |  |  |  |  |  |  |  |  |  |  |
| **H1** | 12.3±3.5 | 11.1±3.6 | 11.9±3.5 | 28.6±6.6 | 14±4.8 | 27.9±3.6 | 53.1±6.7 | 11.6±4.8 |  |  |  |  |  |  |  |  |  |  |  |  |  |  |
| **H2** | 30.7±6.2 | 25±4.7 | 22±5.4 | 22.9±4.3 | 21.6±4.4 | 39.1±7.8 | 70±9.9 | 24.7±3.9 | 12.8±5.4 |  |  |  |  |  |  |  |  |  |  |  |  |  |
| **H3** | 36.3±7.4 | 26.8±3.8 | 29.4±4.7 | 20.4±5.6 | 35.9±7.2 | 38.1±4.7 | 68±7.9 | 26.1±6.5 | 36±7.5 | 55.8±9.5 |  |  |  |  |  |  |  |  |  |  |  |  |
| **I1** | 29.2±6.7 | 18.3±5.9 | 35.1±6.4 | 79.1±11.9 | 45.8±4.4 | 39.4±7.4 | 53.7±4.8 | 21.3±5.2 | 21±4.6 | 47.7±7.6 | 50.4±6.4 |  |  |  |  |  |  |  |  |  |  |  |
| **I2** | 16.5±4.3 | 15.3±6.4 | 11.1±4.3 | 15.6±4.3 | 14.1±6.6 | 9.9±3.3 | 50.3±6.3 | 14±8.4 | 13.4±4.9 | 25.9±5.7 | 38.6±4.7 | 29.3±3.8 |  |  |  |  |  |  |  |  |  |  |
| **M8** | 10.1±3.4 | 11.2±3.7 | 8.4±3.2 | 15.8±3.5 | 28.7±4.7 | 27.4±4.6 | 43.7±5.4 | 19.1±6.5 | 31.3±6.6 | 42.8±3.8 | 24.5±5.5 | 32.3±6.6 | 18.3±6.4 |  |  |  |  |  |  |  |  |  |
| **M9** | 13.4±3.7 | 15.1±4.8 | 14.6±6.5 | 14.4±5.3 | 18±3.9 | 23.4±5.9 | 46.1±5.8 | 13.5±3.6 | 11±3.9 | 22.7±3.9 | 33.3±8.4 | 32.5±4.4 | 19.2±3.5 | 15.3±6.6 |  |  |  |  |  |  |  |  |
| **M10** | 12.7±3.2 | 10.9±3.4 | 11.6±4.2 | 9.8±2.8 | 18±7.4 | 23.4±5.3 | 43±3.9 | 15.6±5.1 | 9.7±2.6 | 31.4±6.6 | 33.7±4.6 | 28.3±3.7 | 14±3.5 | 10.3±5.7 | 13.2±4.4 |  |  |  |  |  |  |  |
| **M21** | 11.7±3.6 | 13.7±3.2 | 22.9±4.5 | 16.8±4.6 | 32.3±5.3 | 19.8±4.7 | 40.6±3.5 | 14.7±3.6 | 13.8±4.7 | 25.8±5.7 | 34.3±3.3 | 47.9±8.6 | 13.5±3.7 | 12.4±4.8 | 29.1±6.5 | 10.7±3.6 |  |  |  |  |  |  |
| **M22** | 14.8±3.6 | 11±3.5 | 16±5.3 | 16.6±3.8 | 15.8±4.6 | 25.6±3.9 | 45.2±13.4 | 11.9±4.7 | 12.6±3.8 | 21.5±3.9 | 35.2±3.3 | 40.8±7.3 | 14.3±5.7 | 21.2±7.8 | 14±3.6 | 14.9±5.5 | 13.5±3.7 |  |  |  |  |  |
| **N2** | 12.2±4.4 | 14.1±5.6 | 11.4±5.3 | 15.9±4.9 | 17.1±4.9 | 21.8±5.3 | 44±6.3 | 13.6±5.9 | 11.8±3.4 | 18.3±6.7 | 36.4±5.6 | 15.3±4.8 | 14±4.3 | 16.3±3.6 | 9.6±2.7 | 10.3±6.3 | 9.9±4.6 | 11±3.3 |  |  |  |  |
| **Q2** | 11±3.6 | 12.3±4.7 | 10.1±4.6 | 22±5.4 | 18.7±6.4 | 25.3±6.9 | 50±4.5 | 12.4±3.7 | 16.7±5.6 | 25.7±8.5 | 41.2±14.9 | 15.5±4.4 | 13.9±3.8 | 13.6±5.8 | 14.4±3.7 | 14.4±4.4 | 11.7±4.9 | 10.9±5.3 | 12.7±4.5 |  |  |  |
| **T1** | 44.8±6.6 | 38.9±5.8 | 47.2±6.7 | 52±3.9 | 22.8±4.5 | 70.1±8.6 | 75.3±6.7 | 47.9±6.9 | 29.3±4.7 | 58.2±7.6 | 74.4±8.8 | 77±8.3 | 34.2±6.6 | 43.7±7.6 | 50.2±7.8 | 40.1±6.5 | 51.1±5.7 | 44.7±4.5 | 28.3±3.7 | 29.6±8.3 |  |  |
| **T2** | 16.4±5.2 | 16±4.4 | 20.1±5.2 | 17.2±4.5 | 16.3±4.2 | 18.7±3.3 | 33.2±4.8 | 11±3.6 | 11.5±3.8 | 17.1±3.5 | 34.8±4.6 | 35.1±9.5 | 12±3.6 | 12.9±5.3 | 21.5±5.4 | 12.6±4.5 | 14.1±4.5 | 12.8±5.2 | 12.7±6.6 | 14.6±5.2 | 48.7±5.6 |  |
