# Supplementary material for: Isolation and Characterization of Broad Spectrum Coaggregating Bacteria from Different Water Systems for Potential Use in Bioaugmentation
Source: PLoS One. 2014 Apr 15;9(4):e94220. doi: 10.1371/journal.pone.0094220 (PMC3988075; doi:10.1371/journal.pone.0094220)
Supplement: Table S2 — Coaggregation index (A.I.) for bacterial pairs from 22 strains at 20 h. The bacteria were incubated at 20°C; the OD660 of the supernatant were measured. The values are the average of three independent experiments with deviation score in brackets. (DOCX) [file pone.0094220.s002.docx]

| **Strain** | | | | | | | | | | | | | | | | | | | | | | |  |
| --- | --- | --- | --- | --- | --- | --- | --- | --- | --- | --- | --- | --- | --- | --- | --- | --- | --- | --- | --- | --- | --- | --- | --- |
| **Strain** | **A3** | **XCZ** | **DLL** | **F2** | **F3** | **G3** | **G5** | **G6** | **H1** | **H2** | **H3** | **I1** | **I2** | **M8** | **M9** | **M10** | **M21** | **M22** | **N2** | **Q2** | **T1** | **T2** | |
| **A3** |  |  |  |  |  |  |  |  |  |  |  |  |  |  |  |  |  |  |  |  |  |  | |
| **XCZ** | 18.2±5.6 |  |  |  |  |  |  |  |  |  |  |  |  |  |  |  |  |  |  |  |  |  | |
| **DLL-1** | 18.7±4.7 | 16.4±4.3 |  |  |  |  |  |  |  |  |  |  |  |  |  |  |  |  |  |  |  |  | |
| **F2** | 46.6±7.5 | 40.4±6.1 | 45.0±5.4 |  |  |  |  |  |  |  |  |  |  |  |  |  |  |  |  |  |  |  | |
| **F3** | 26.2±4.6 | 42.3±4.4 | 49.5±3.5 | 38.3±4.5 |  |  |  |  |  |  |  |  |  |  |  |  |  |  |  |  |  |  | |
| **G3** | 37.7±7.6 | 49.5±7.2 | 31.2±6.3 | 43.1±7.6 | 49.0±3.6 |  |  |  |  |  |  |  |  |  |  |  |  |  |  |  |  |  | |
| **G5** | 57.2±8.4 | 59.3±3.5 | 50.6±6.7 | 67.9±4.5 | 58.3±7.6 | 80.6±12.5 |  |  |  |  |  |  |  |  |  |  |  |  |  |  |  |  | |
| **G6** | 24.8±4.7 | 27.3±5.7 | 26.0±3.8 | 31.8±7.7 | 36.5±6.7 | 27.5±5.4 | 53.2±6.2 |  |  |  |  |  |  |  |  |  |  |  |  |  |  |  | |
| **H1** | 18.4±3.4 | 20.0±3.0 | 19.5±4.3 | 49.2±11.3 | 24.8±4.4 | 55.8±8.6 | 72.0±7.4 | 22.9±5.7 |  |  |  |  |  |  |  |  |  |  |  |  |  |  | |
| **H2** | 24.8±4.7 | 26.4±10.6 | 31.2±6.5 | 57.1±5.4 | 24.0±6.2 | 56.5±6.7 | 77.6±6.6 | 37.0±7.5 | 17.7±4.7 |  |  |  |  |  |  |  |  |  |  |  |  |  | |
| **H3** | 44.1±8.5 | 39.1±4.6 | 40.1±4.4 | 64.5±7.3 | 32.4±4.6 | 57.6±5.5 | 76.2±5.8 | 38.4±5.4 | 26.2±6.5 | 53.9±6.5 |  |  |  |  |  |  |  |  |  |  |  |  | |
| **I1** | 37.7±4.2 | 82.3±10.5 | 45.1±6.7 | 87.3±5.4 | 71.6±11.0 | 54.9±6.8 | 55.0±7.5 | 32.9±6.8 | 40.0±5.3 | 56.4±7.4 | 60.8±6.4 |  |  |  |  |  |  |  |  |  |  |  | |
| **I2** | 23.0±6.6 | 23.6±4.6 | 24.3±4.8 | 38.4±7.6 | 18.6±6.4 | 22.0±4.3 | 63.5±8.7 | 17.6±3.6 | 27.6±4.6 | 30.2±5.4 | 41.4±3.6 | 32.3±8.6 |  |  |  |  |  |  |  |  |  |  | |
| **M8** | 16.8±5.9 | 18.0±3.7 | 22.9±5.4 | 12.1±4.7 | 27.3±4.5 | 27.7±6.7 | 52.4±5.4 | 26.6±6.5 | 21.7±4.8 | 35.0±5.7 | 34.1±4.3 | 20.4±9.3 | 29.6±5.6 |  |  |  |  |  |  |  |  |  | |
| **M9** | 26.2±8.4 | 23.5±3.3 | 33.6±4.3 | 25.6±5.5 | 24.0±6.6 | 32.0±6.4 | 56.6±4.7 | 23.5±5.7 | 16.4±6.7 | 44.1±6.5 | 38.0±6.5 | 32.3±5.3 | 20.6±3.5 | 52.6±13.3 |  |  |  |  |  |  |  |  | |
| **M10** | 16.8±5.7 | 15.0±6.5 | 18.4±5.2 | 51.0±9.4 | 24.9±3.6 | 26.9±4.5 | 48.5±6.5 | 23.4±6.4 | 17.0±6.5 | 10.6±3.5 | 40.9±4.6 | 39.9±4.6 | 13.2±2.8 | 11.1±6.5 | 51.0±4.3 |  |  |  |  |  |  |  | |
| **M21** | 22.1±7.3 | 17.9±5.3 | 29.6±6.5 | 37.4±6.9 | 30.9±5.7 | 26.4±3.6 | 47.6±5.6 | 22.3±5.6 | 12.8±4.7 | 39.4±6.3 | 45.4±8.4 | 55.5±7.4 | 17.8±3.8 | 11.1±4.7 | 91.9±16.5 | 19.9±5.3 |  |  |  |  |  |  | |
| **M22** | 21.2±3.3 | 16.2±4.6 | 20.8±4.7 | 47.8±6.3 | 32.0±6.3 | 27.2±6.4 | 53.7±3.7 | 24.3±4.7 | 22.4±6.6 | 24.7±4.3 | 40.7±5.4 | 45.1±5.5 | 21.3±5.7 | 21.3±5.5 | 50.2±6.7 | 15.3±4.4 | 15.0±5.5 |  |  |  |  |  | |
| **N2** | 15.3±4.6 | 15.4±3.7 | 15.1±3.9 | 37.5±7.2 | 15.2±9.4 | 28.7±6.6 | 50.7±7.6 | 20.9±6.4 | 19.6±6.8 | 23.6±5.6 | 39.2±7.2 | 18.1±3.6 | 20.2±6.3 | 16.3±4.7 | 12.6±3.6 | 10.6±3.7 | 14.5±3.6 | 11.0±4.2 |  |  |  |  | |
| **Q2** | 16.4±6.6 | 18.1±5.7 | 12.5±3.6 | 31.7±6.3 | 16.8±5.6 | 31.2±7.2 | 55.6±6.7 | 23.5±4.2 | 24.1±3.8 | 40.0±9.7 | 58.2±12.4 | 21.2±2.9 | 23.3±4.4 | 27.0±4.3 | 14.9±3.8 | 13.5±4.9 | 15.9±6.5 | 13.2±6.4 | 16.7±6.3 |  |  |  | |
| **T1** | 60.8±6.4 | 45.2±8.4 | 54.2±6.3 | 78.2±5.7 | 82.4±7.7 | 81.4±8.5 | 85.7±8.4 | 54.0±8.4 | 34.9±6.6 | 64.0±7.8 | 81.8±5.6 | 81.9±8.5 | 42.0±4.8 | 65.2±7.5 | 64.3±7.4 | 53.3±6.5 | 60.3±8.6 | 54.6±7.6 | 33.4±4.4 | 36.0±5.5 |  |  | |
| **T2** | 23.3±4.2 | 16.8±5.3 | 23.8±4.4 | 40.6±7.6 | 43.0±8.3 | 47.3±5.6 | 53.8±4.5 | 28.5±6.5 | 15.1±3.4 | 23.4±4.4 | 40.9±4.8 | 36.8±7.6 | 22.4±3.9 | 11.5±3.5 | 65.7±13.8 | 13.7±5.3 | 31.9±4.4 | 16.8±3.4 | 8.1±2.7 | 17.0±4.7 | 63.7±4.3 |  | |

**Table S2 Coaggregation index ( A.I) for bacterial pairs from 22 strains at 20h**
